# Transparency of high‐dimensional propensity score analyses: Guidance for diagnostics and reporting

**DOI:** 10.1002/pds.5412

**Published:** 2022-02-12

**Authors:** John Tazare, Richard Wyss, Jessica M. Franklin, Liam Smeeth, Stephen J. W. Evans, Shirley V. Wang, Sebastian Schneeweiss, Ian J. Douglas, Joshua J. Gagne, Elizabeth J. Williamson

**Affiliations:** ^1^ Faculty of Epidemiology and Population Health London School of Hygiene and Tropical Medicine London UK; ^2^ Division of Pharmacoepidemiology and Pharmacoeconomics Brigham and Women's Hospital and Harvard Medical School Boston Massachusetts USA; ^3^ Health Data Research (HDR) UK London UK

**Keywords:** confounder adjustment, database research, diagnostics, high dimensional propensity score, reporting

## Abstract

**Purpose:**

The high‐dimensional propensity score (HDPS) is a semi‐automated procedure for confounder identification, prioritisation and adjustment in large healthcare databases that requires investigators to specify data dimensions, prioritisation strategy and tuning parameters. In practice, reporting of these decisions is inconsistent and this can undermine the transparency, and reproducibility of results obtained. We illustrate reporting tools, graphical displays and sensitivity analyses to increase transparency and facilitate evaluation of the robustness of analyses involving HDPS.

**Methods:**

Using a study from the UK Clinical Practice Research Datalink that implemented HDPS we demonstrate the application of the proposed recommendations.

**Results:**

We identify seven considerations surrounding the implementation of HDPS, such as the identification of data dimensions, method for code prioritisation and number of variables selected. Graphical diagnostic tools include assessing the balance of key confounders before and after adjusting for empirically selected HDPS covariates and the identification of potentially influential covariates. Sensitivity analyses include varying the number of covariates selected and assessing the impact of covariates behaving empirically as instrumental variables. In our example, results were robust to both the number of covariates selected and the inclusion of potentially influential covariates. Furthermore, our HDPS models achieved good balance in key confounders.

**Conclusions:**

The data‐adaptive approach of HDPS and the resulting benefits have led to its popularity as a method for confounder adjustment in pharmacoepidemiological studies. Reporting of HDPS analyses in practice may be improved by the considerations and tools proposed here to increase the transparency and reproducibility of study results.

**Key Points**

- The high‐dimensional propensity score (HDPS) is a well‐established method for variable identification, prioritisation, and adjustment tailored for use in large healthcare databases.
- Diagnostic tools can offer important insights into the properties of the features selected for inclusion in HDPS models.
- We provide considerations for reporting to increase the transparency and reproducibility of HDPS analyses.
- We hope more widespread use of the guidance and tools presented will help to breakdown ‘black‐box’ criticisms of the HDPS.

**Plain Language Summary**

The high‐dimensional propensity score (HDPS) algorithm is an established method for identifying and selecting confounders in large healthcare databases. The implementation of HDPS approaches can be complex, requiring investigators to identify data for consideration and specify tuning parameters. We present graphical tools and sensitivity analyses for evaluating HDPS analyses and discuss key reporting considerations to help promote transparent implementation of HDPS approaches. These are illustrated using a study of proton pump inhibitor users from the UK Clinical Practice Research Datalink. Seven items for reporting key aspects of the HDPS procedure were identified, including clear specification of the types of input data considered and the number of variables selected by the HDPS. Graphical visualization tools included summaries of the types and properties of variables selected by the HDPS. Sensitivity analyses included investigation of the robustness of results to the key decision surrounding number of variables selected. We hope the tools and considerations provided allow researchers to better scrutinize HDPS analyses and improve the reporting of implementation details.

## 1 INTRODUCTION

Bias arising from confounding is a key concern for pharmacoepidemiological studies and its mitigation depends on the ability to identify, measure and adjust for underlying differences between patients receiving different therapies.^1^ Successful adjustment for confounding often hinges on capturing hard to measure concepts, such as markers of frailty, disease severity, or health‐seeking behaviour.

The high‐dimensional propensity score (HDPS) algorithm^2^, ^3^ is a method for variable identification, prioritisation, and adjustment tailored for large healthcare databases. The HDPS conceptualises information in these databases as proxies to key underlying constructs; some are likely to be strongly correlated with other measured variables, but others act as proxies for constructs that would otherwise be unmeasured. The procedure treats these features as additional covariates for adjustment with the aim of optimising confounding capture and control.

Whilst the HDPS often incorporates several hundred additional covariates, the types of features included are rarely communicated leading some to label the HDPS a ‘black‐box’ approach. Diagnostic tools can offer important insights into the properties of these features, enhancing our knowledge of the factors driving treatment decisions and checking for possible errors, e.g., the presence of certain codes in the pool of selected HDPS covariates can highlight possible errors relating to linkage error or the application of exclusion criteria.

Despite studies highlighting the potential lack of robustness to investigator decisions (e.g., the number of covariates chosen),^4^, ^5^ reporting of sensitivity analyses remains inconsistent and this can undermine the transparency and reproducibility of HDPS analyses. Recent guidelines surrounding the reporting of pharmacoepidemiological studies state that “high dimensional proxy adjustment” methods should be reported in full; guidance is needed about what this entails.^6^

Building on existing guidance for propensity score (PS) analysis,^7^, ^8^, ^9^ we describe and illustrate diagnostic tools and sensitivity analyses for HDPS analyses. We also provide considerations for reporting relevant information.

## 2 HIGH‐DIMENSIONAL PROPENSITY SCORES

The generic five steps of the HDPS procedure are as follows^2^:

- Step one, investigators specify the data structure. This can involve declaring data dimensions capturing different aspects of care in the database under investigation.
- Step two, pre‐exposure features are generated, and a prevalence filter is typically applied (often selecting the top 200 most common features from each dimension). Features are usually in the form of codes or free‐text information and grouped at a specific granularity level. For example, codes might be truncated to the first three digits if they are International Classification of Diseases, 10th edition (ICD‐10) codes.
- Step three, the recurrence of features is assessed in a pre‐exposure period, creating binary covariates based on a set of frequency‐based cut‐offs.^2^ The standard implementation of the HDPS defines three indicators for each patient capturing whether a feature was recorded: ≥once, ≥the median, and ≥the 75th percentile.
- Step four, the large pool of covariates generated in the previous step is prioritised. This is typically achieved using the Bross formula, which uses univariate associations of covariates with treatment and outcome, to identify those with the highest potential to bias the treatment‐outcome relationship.^2^, ^3^
- Step five, a number of HDPS covariates (typically the top 200–500 from the covariate prioritisation)^2^, ^10^ are selected to augment a set of pre‐defined variables (selected by the investigators based on background knowledge) used for estimation of the PS model. Standard PS methods (e.g. matching or weighting)^8^, ^11^ are used to estimate treatment effects based on both sets of covariates. The guidance presented subsequently should be considered additional to existing practices surrounding the reporting of PS methods e.g., summarising weights by exposure groups or presenting the proportion of patients unmatched.^7^, ^8^

## 3 CONSIDERATIONS FOR REPORTING

We initially conducted a literature search surrounding PS diagnostics and reporting guidance, identifying important gaps in the current literature surrounding the reporting of HDPS models. Utilising the extensive experience and knowledge of HDPS analyses within the research team, we present considerations for reporting features of the HDPS procedure (summarised in Table 1).

**TABLE 1:** Reporting considerations for key features and decisions of the high‐dimensional propensity score approach

Item	Description	Aspect(s) to report
1	Specify data dimensions	Dimensions identified and which aspect of the healthcare system they characterise
2	Describe parameters for generating pre‐exposure features	Describe how features are generated
		Number of codes selected per dimension in prevalence filter
3	Describe feature recurrence assessment	Whether and how recurrence was considered
		Whether and how proximity to exposure start was considered
4	Specify covariate prioritisation method	Ranking based on:
		‐ Exposure‐outcome prediction based (Bross)
		‐ ML‐supported exposure‐outcome prediction
		‐ Exposure prediction only
5	Specify total number of covariates to select	Number of HDPS covariates selected
		Justification for number of HDPS covariates selected, e.g. use of simulation‐based approaches
		Routine reporting of the investigator identified covariates
6	Specify software	Describe which software package was used to implement the HDPS procedure
7	Describe the results of diagnostics and sensitivity analyses	Describe diagnostic tools used and highlight key insights gained
		Describe the results of sensitivity analyses and discuss the possible implications for interpreting the findings from the primary analysis

### Item 1: Specify data dimensions

Data dimensions identified should be summarised, indicating which aspects of care they capture and possibly note data quality and completeness metrics. These summaries should include a description of the features included in the data dimensions (e.g. codes, free‐text information, laboratory test results) and any corresponding coding systems used.

### Item 2: Describe parameters for generating pre‐exposure features

Investigators should describe how features are generated, e.g. specifying the code granularity for a particular coding system (e.g. 3‐digit ICD‐10) or how free‐text information has been processed.^12^ Furthermore, investigators may describe the number of candidate features available within the specified dimensions.

Ongoing debate in the literature surrounds the use of marginal prevalence for prioritising features in Step 2 of the HDPS procedure.^13^ The main concern is the possible omission of influential features where despite a low marginal prevalence there exist strong imbalances within exposure groups. Investigators should indicate whether the prevalence filter is used and if so, state the number of features selected per dimension.

### Item 3: Describe feature recurrence assessment

Whilst feature recurrence is typically assessed using the cut‐offs outlined by Schneeweiss et al, deviations from these cut‐offs exist and should be described in full.^10^, ^14^ One example suggests explicitly considering the proximity to exposure start.^10^

### Item 4: Specify covariate prioritization method

Investigators should describe the method of covariate prioritisation used. Whilst ranking is typically based on the Bross formula, exposure‐based ranking (prioritising covariates based on the confounder‐exposure association) has been employed in settings with few outcome events.^2^, ^5^

Recent evidence indicates the potential for machine‐learning methods to enhance the performance of HDPS, both for covariate prioritisation or by reducing the set of covariates prioritised by the Bross formula.^15^, ^16^, ^17^

### Item 5: Specify total number of HDPS covariates to select

The number of HDPS covariates selected for inclusion in the PS model should be reported, in addition to routine reporting of the investigator‐identified covariates. Machine learning‐based approaches to determine the number of codes selected should be described in full.^4^, ^5^, ^17^

### Item 6: Specify software

Investigators should describe which software was used to implement the HDPS. There are commonly used packages available in R,^18^ SAS,^19^ or Aetion.

### Item 7: Describe the results of diagnostics

Subsequent sections describe and discuss the interpretation of relevant diagnostic tools and sensitivity analyses that should be routinely conducted and reported.

## 4 DATA FOR ILLUSTRATION

### 4.1 Background

We use a cohort study from the United Kingdom (UK) Clinical Practice Research Datalink (CPRD) linked with the Myocardial Ischaemia National Audit Project (MINAP).^20^ The CPRD is a database capturing information pertaining to contacts with primary care services (including clinical diagnoses, referrals and prescriptions) and is broadly representative of patients registered at general practitioners in the UK.^21^

The study investigated whether a pharmacokinetic interaction between clopidogrel and use of proton pump inhibitors (PPI) could reduce clopidogrel effectiveness, leading to increased risk of vascular events. Results indicated an increased risk of MI associated with PPI use, which was hypothesised to be largely due to residual confounding between treatment groups.^20^

A reanalysis using the HDPS obtained results much closer to the hypothesised null association,^20^, ^22^, ^23^ suggesting an improved ability to account for between‐patient characteristics that were important for confounding control.^14^

### 4.2 Summary of HDPS analysis

We defined three dimensions assessing clinical, referral, and therapy information in the year prior to cohort entry. We applied a prevalence filter selecting the top 200 features from each dimension and adjusted for the top 500 HDPS covariates (prioritised by the Bross formula).^14^

The PS was estimated using multivariable logistic regression including both pre‐defined and HDPS covariates. Hazard ratios (HR) for the treatment effect were obtained using Cox regression weighted by inverse probability of treatment weights. Standard errors for treatment effects were obtained using robust standard errors.^14^

Table 2 summarises the results, including a sensitivity analysis varying the number of HDPS covariates selected.

**TABLE 2:** Summary of Clinical Research Practice Datalink study used for illustration investigating the association between proton pump inhibitor use and risk of myocardial infarction in a population of clopidogrel and aspirin users

Analysis	Number of covariates	Hazard ratio (95% CI)
Crude	0	1.23 (1.06–1.42)
Pre‐defined only^a^	10	1.17 (1.00–1.35)
Primary HDPS	10 + 500	1.00 (0.78–1.28)
Sensitivity	10 + 100	1.07 (0.87–1.32)
	10 + 250	1.02 (0.81–1.27)
	10 + 750	1.03 (0.79–1.28)

^a^ Pre‐defined covariates: age, sex, smoking status, alcohol status, categorised BMI, alcohol status, history of PVD, CHD, stroke and cancer.

Analyses were conducted using Stata 15 and R.^24^, ^25^ Code reproducing the figures presented is available at www.github.com/johntaz/HDPS-Diagnostics.

## 5 DIAGNOSTIC AND VISUALISATION TOOLS

In this section, we illustrate and discuss novel and established PS diagnostics for assessing HDPS models (summarised in Table 3).

**TABLE 3:** Summary of established and proposed diagnostic tools for high‐dimensional propensity score models

Diagnostic description	Section discussed	Conventional propensity score	High‐dimensional propensity score
Propensity score distribution by treatment group	5.2	**✓**	**✓**
Prevalence of selected covariates by treatment group	5.3	–	**✓**
Absolute standardised differences	5.3	**✓**	**✓**
Bross‐derived prioritisation distribution	5.4	–	**✓**
Relationship between confounder‐exposure and confounder‐outcome associations	5.4	–	**✓**

### 5.1 Model summaries

**FIGURE 1:**
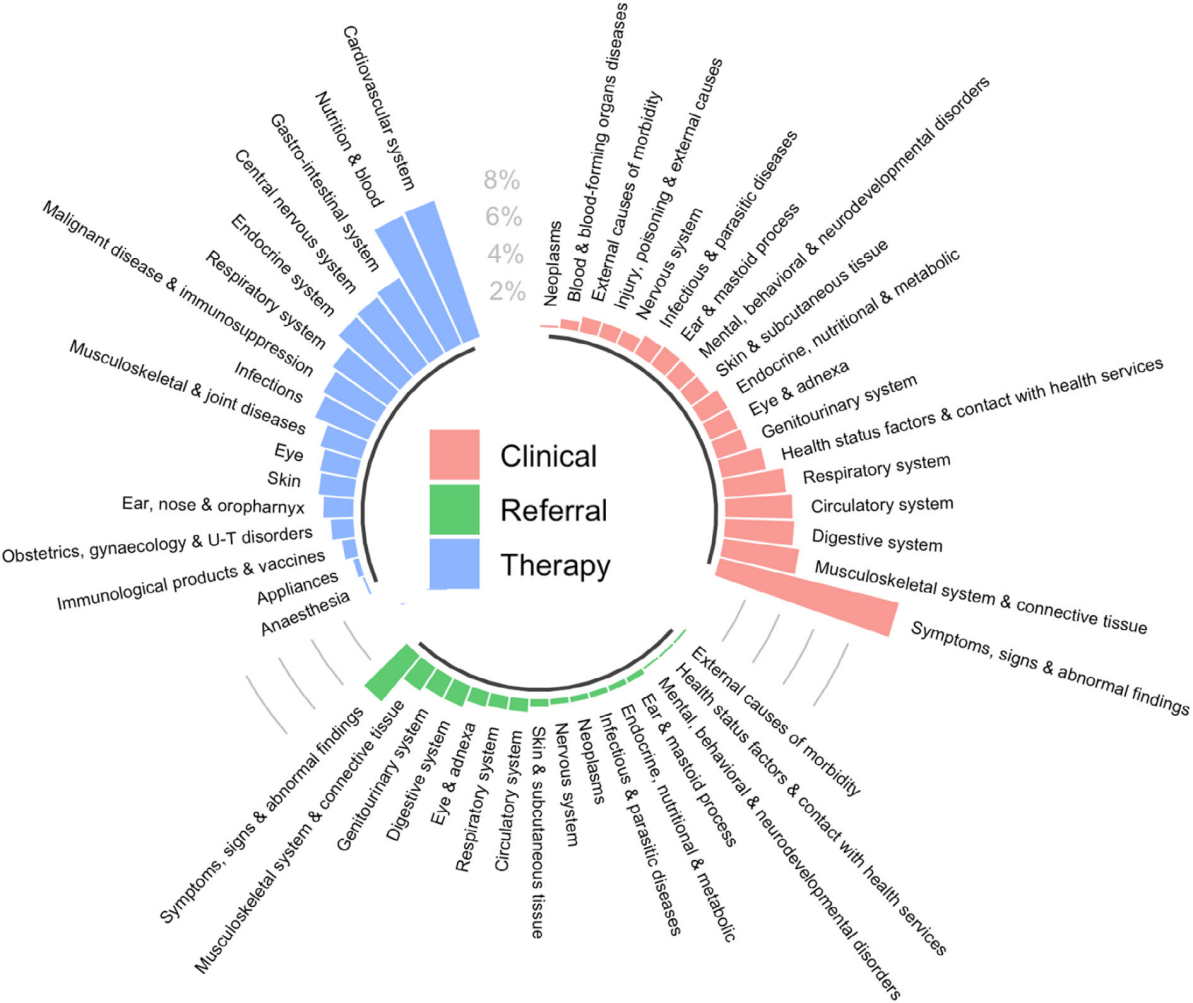
Summary of high‐level concepts captured in the top 750 Bross‐prioritised high‐dimensional propensity score pre‐exposure covariates separated and colour‐coded by data dimension

We recommend simple descriptions for communicating the covariates included in HDPS models, e.g., highlighting the proportion of selected codes that came from each data dimension. Investigators may also summarise high‐level clinical concepts captured by the covariates included in the HDPS. Our study categorised codes using British National Formulary (BNF) paragraph level (prescription dimension) and ICD‐10 (clinical and referral dimensions). We exploited the hierarchy of these coding systems to investigate codes aggregated by the chapter level. Figure 1 shows that in the clinical and referral dimensions, the majority of covariates selected corresponded to codes relating to symptoms, signs and abnormal findings. Additionally, covariates derived from the therapy dimension corresponded most to prescriptions from the cardiovascular system or nutrition and blood BNF chapters.

### 5.2 Comparison of PS distributions

Inspecting the distributions of the estimated PS by treatment group is a common diagnostic highlighting the ability of covariates included in the PS model to predict treatment received in the population being studied. As with all PS analyses, investigators should verify the positivity assumption,^9^ a violation of which is lack of overlap. One common approach for handling this is PS trimming.^26^, ^27^

**FIGURE 2:**
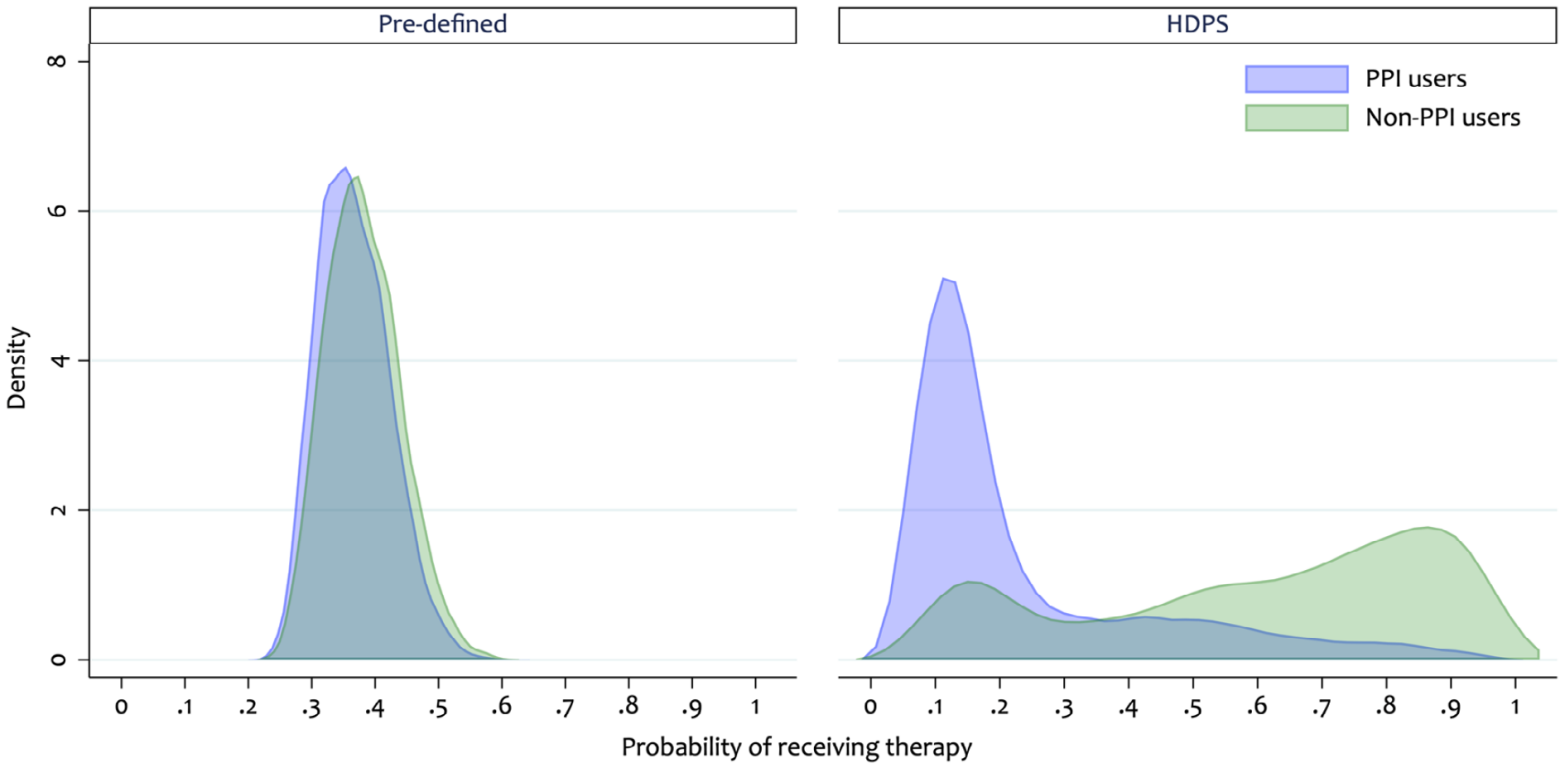
Overlap plot comparing the propensity score distributions including only 10 pre‐defined pre‐exposure covariates and additionally including the 500 top‐ranked high‐dimensional propensity score covariates

Whilst inspection of the estimated PS distribution is recommended when applying the HDPS, it is additionally informative to compare the PS distributions before and after inclusion of the HDPS covariates. This requires estimating the PS under models including a) only the pre‐defined covariates and b) the pre‐defined and selected HDPS covariates. Figure 2 compares the estimated PS distributions under these models.

When including only the pre‐defined covariates, the estimated PS distributions appear similar between the treatment groups (Figure 2).^28^ However, when adding the HDPS covariates we observe a shift in the PS distributions (Figure 2), indicating that, in this example, the HDPS has captured extra predictors of treatment initiation. This highlights important between‐patient differences not apparent when only including the pre‐defined covariates. These differences would not be accounted for under the investigator‐led PS analysis.

### 5.3 Covariate balance

**FIGURE 3:**
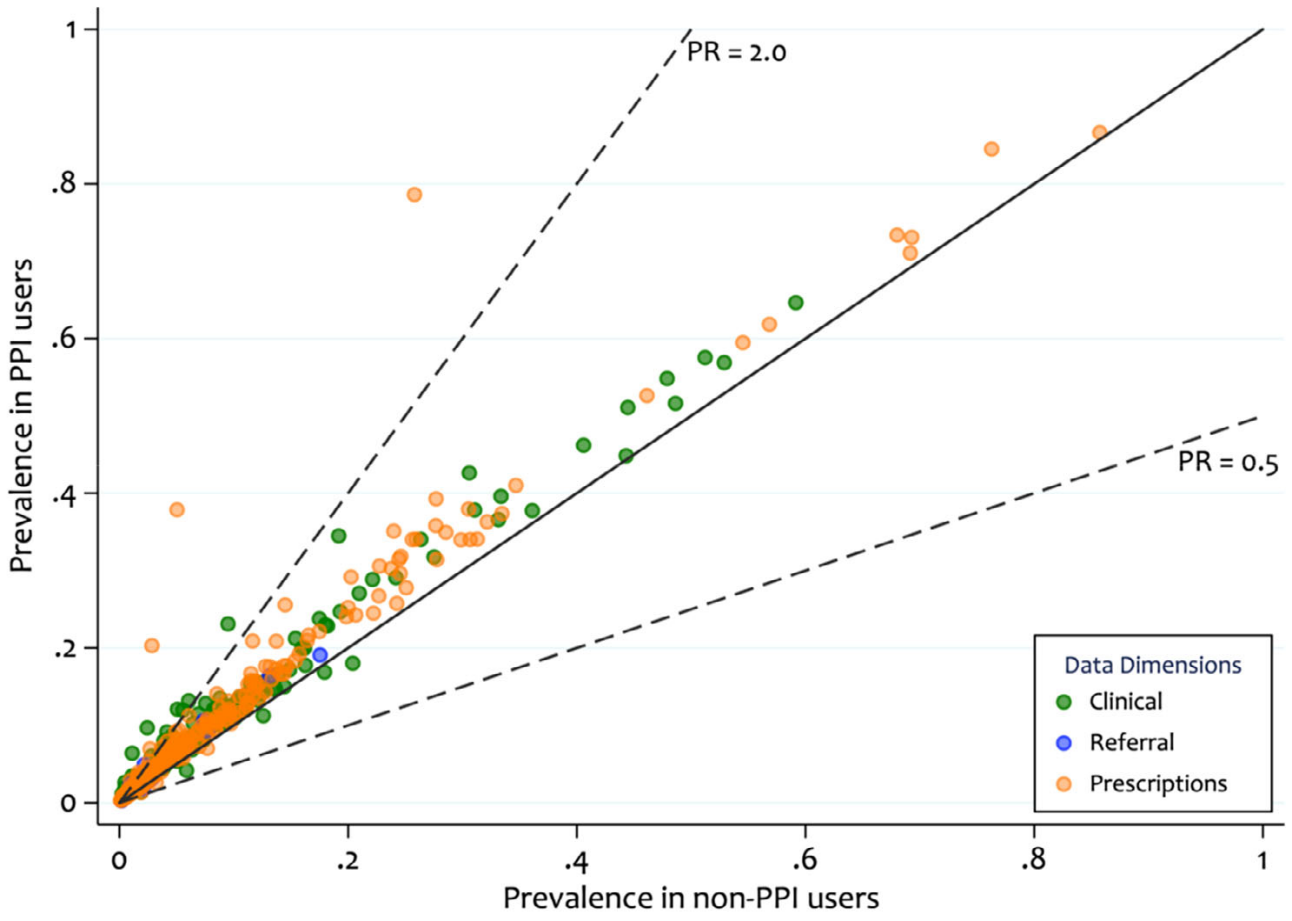
Prevalence of the top 500 Bross‐prioritised high‐dimensional propensity score pre‐exposure covariates by treatment group and by data dimension. The diagonal line indicates equal prevalence in both groups and the dashed lines show prevalence ratios (PR) of 0.5 and 2.0. The colour coding highlights, which dimension the covariate, was derived from

To investigate the overall balance of HDPS covariates we can plot the prevalence of selected covariates between the two treatment groups (shown in Figure 2).^28^ Figure 3 highlights that for most covariates there is a similar prevalence in both groups, with slightly higher prevalence amongst the PPI users. There are several covariates from the prescription dimension (Figure 3, prevalence ratio >2.0) with moderate to high prevalence amongst PPI users and a low prevalence amongst the non‐users.

**FIGURE 4:**
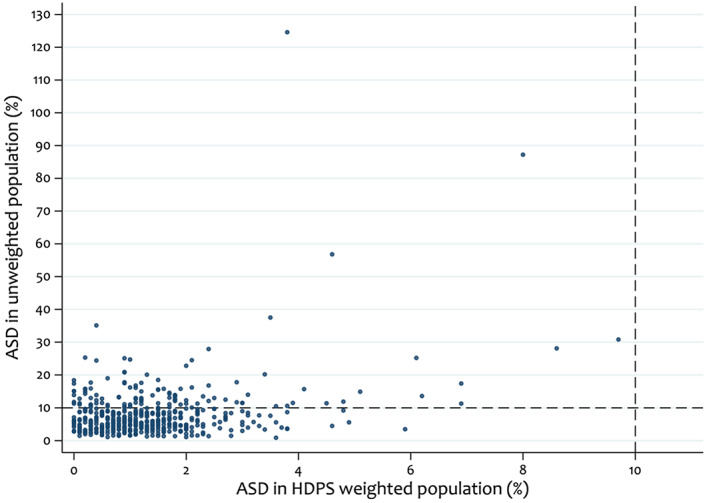
Comparison of absolute standardised differences (ASDs) between unweighted and high‐dimensional propensity score (HDPS) weighted sample under the primary analysis, selecting the top 500 HDPS covariates. Dashed lines indicate absolute standardised differences of 10%

Measures of covariate balance (e.g. absolute standardised differences) are commonly used when assessing PS models to check for imbalances. In the HDPS setting, investigators should check the balance in the HDPS covariates before and after adjustment. Figure 4 indicates some covariates with large imbalances (substantially >10%) in the unweighted population but all achieve good balance in the HDPS weighted population.

There is a concern that adjusting for many additional HDPS confounders can make achieving balance in pre‐defined confounders more difficult, as the PS model tries to simultaneously balance many more variables. If the HDPS variables are weak confounders or even not true confounders, addition of these variables can result in unnecessarily increased bias and variance.^29^, ^30^ Achieving balance is more important in strong confounders compared to weak confounders.^31^ Therefore, we recommend assessing the balance on selected key confounders before and after inclusion of all selected HDPS covariates.^32^

**FIGURE 5:**
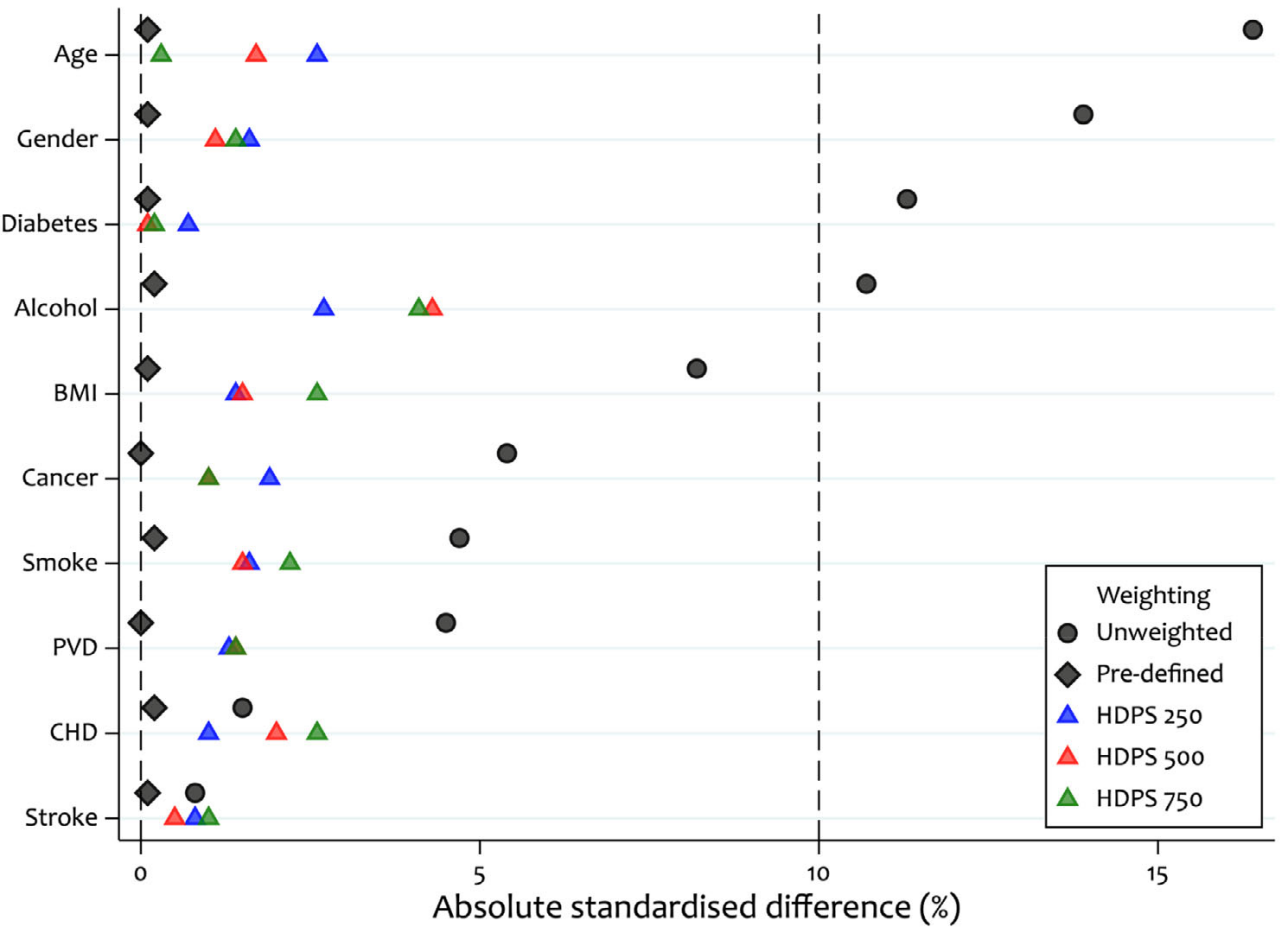
Comparison of absolute standardised differences in a set of key covariates between unweighted, pre‐defined covariate weighted, and pre‐defined and high‐dimensional propensity score covariate weighted samples

For illustrative purposes, we assume that all pre‐defined covariates are important confounders and Figure 5 presents the balance of these covariates under models additionally including 250, 500 and 750 HDPS covariates. We observe that even after adjusting for 750 HDPS covariates, we achieve good balance in the pre‐defined covariates, indicating the suitability of any of these models for preserving balance in the pre‐defined covariates.

**FIGURE 6:**
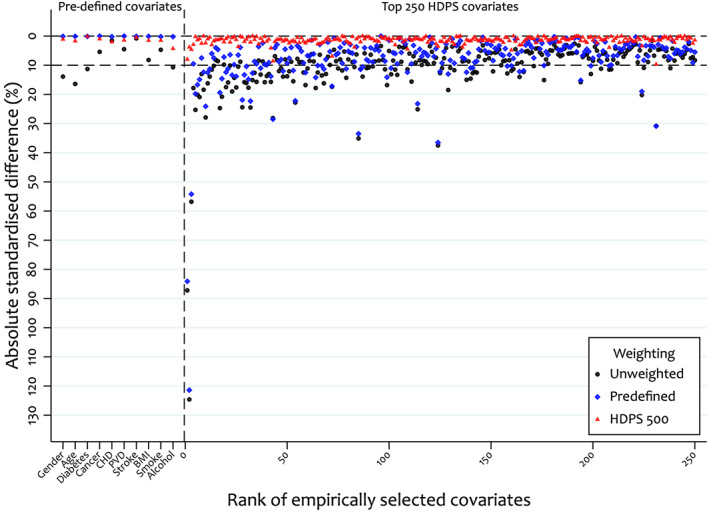
Comparison of absolute standardised differences in the pre‐defined and top 250 high‐dimensional propensity score covariates between unweighted, pre‐defined and HDPS (+500 covariates) weighted samples

Another approach investigates the covariate balance in both the pre‐defined and a set of key HDPS confounders (Figure 6); we additionally assume all key HDPS confounders are in the top 250. Figure 6 highlights that in the pre‐defined weighted population, a number of the top‐ranked HDPS covariates remain imbalanced. However, when weighting by our primary HDPS model we achieve good balance in both the pre‐defined and top 250 covariates.

In Table 4, we present mean absolute standardised differences to measure overall covariate balance. For the pre‐defined covariates, we observe an increase in imbalance when additionally accounting for the HDPS covariates and this is similar under all HDPS models. Furthermore, we observe that when considering all key confounders (pre‐defined and HDPS) the HDPS models perform similarly and achieve better balance than the pre‐defined model. In this study, there is little difference in overall balance between the HDPS models, however other studies might see a deterioration in overall balance when including more HDPS covariates. Overall summaries of imbalance could be modified to put more weight on imbalance in covariates thought to be stronger confounders (in which imbalance is more likely to result in confounding bias); Table 4 presents one method for achieving this.

**TABLE 4:** Comparison of the mean absolute standardised differences in the unweighted, pre‐defined, and pre‐defined and high‐dimensional propensity score (HDPS) weighted populations

Set of covariates	Accounting for relative importance of HDPS covariates^a^	Mean absolute standardised differences				
		Unweighted	Pre‐defined only weighted	Top 250 HDPS weighted	Top 500 HDPS weighted	Top 750 HDPS weighted
Pre‐defined only	–	7.74	0.11	1.56	1.51	1.68
Top 250 HDPS only	No	10.91	8.15	1.14	1.42	1.51
	Yes	6.73	5.11	0.62	0.77	0.88
Pre‐defined and top 250 HDPS	No	10.79	7.84	1.14	1.43	1.51
	Yes^b^	6.77	4.92	0.64	0.80	0.83

^a^ Given a ranked (e.g., Bross‐formula ranking) set of HDPS covariates of size *N*, importance weights are defined as ([*N* + 1] − rank)/*N*.

^b^ Predefined covariates are assigned an importance weight of 1.

The HDPS aims to optimise confounder adjustment but there is a potential trade‐off between better adjustments for a broader array of potential confounders versus tighter balance on key confounders. How much imbalance we are willing to permit in key confounders is primarily driven by how strongly these confounders are associated with the outcome. Therefore, a lack of imbalance in pre‐defined and HDPS covariates does not necessarily mean all confounding has been removed and key unmeasured confounders may still exist.

### 5.4 Identification of potentially influential covariates

Whilst the full list of covariates selected is sometimes provided,^2^ this is not easily digestible when interrogating several hundred HDPS covariates. However, manually inspecting the top covariates included can identify groups of codes relating to previously overlooked concepts that are important for minimising confounding bias.^33^

**FIGURE 7:**
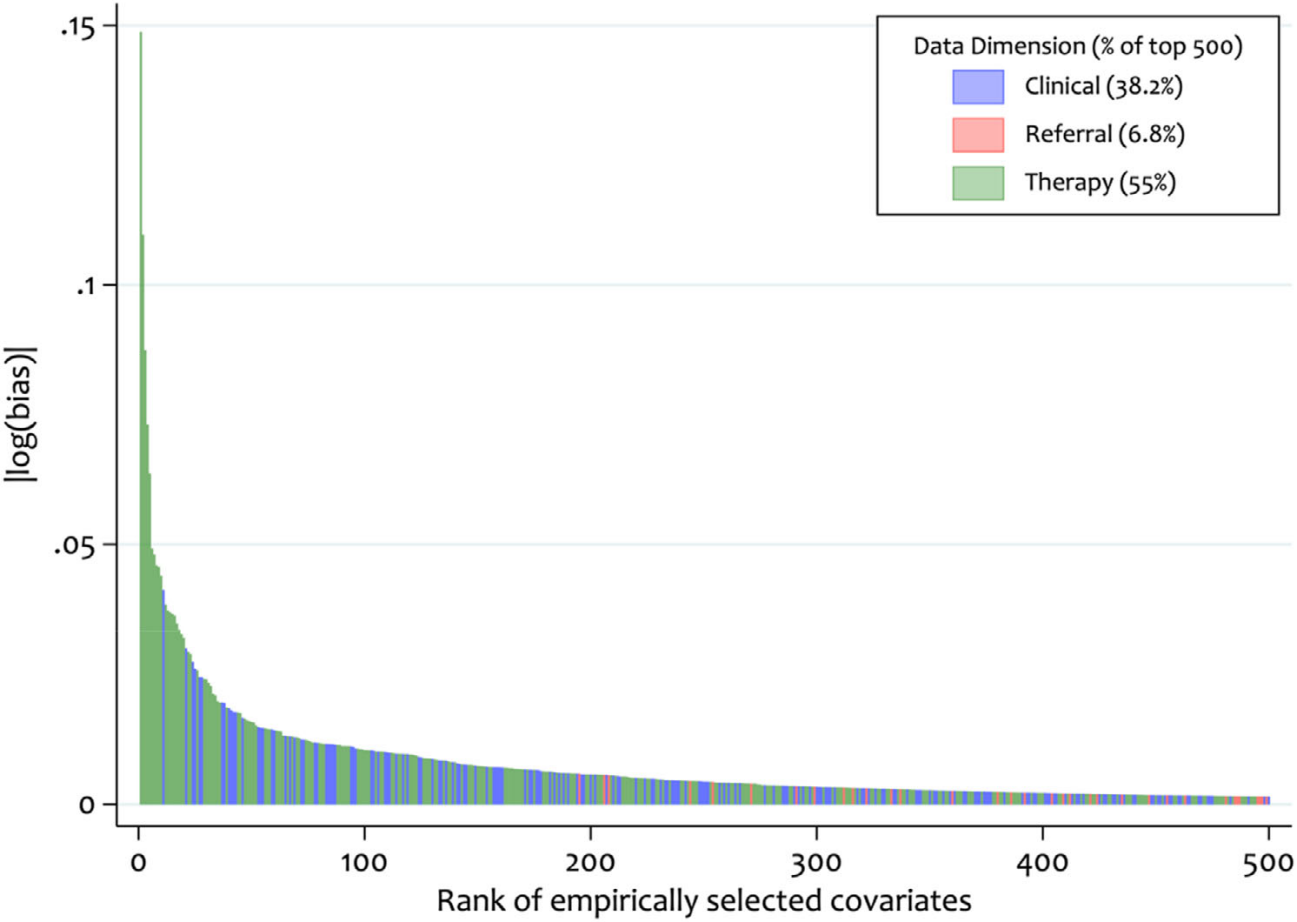
Distribution of absolute log Bross bias values for each of the top 500 high‐dimensional propensity score pre‐exposure covariates

An initial step is to investigate the distribution of Bross‐derived bias values; Figure 7 shows the ranking score for the top 500 covariates.^4^ The colour coding indicates which dimension the covariates originated from and highlights that the majority of covariates were from the prescription dimension. Furthermore, this plot allows investigators to observe highly ranked covariates, which might have a large amount of influence in the PS model.

**FIGURE 8:**
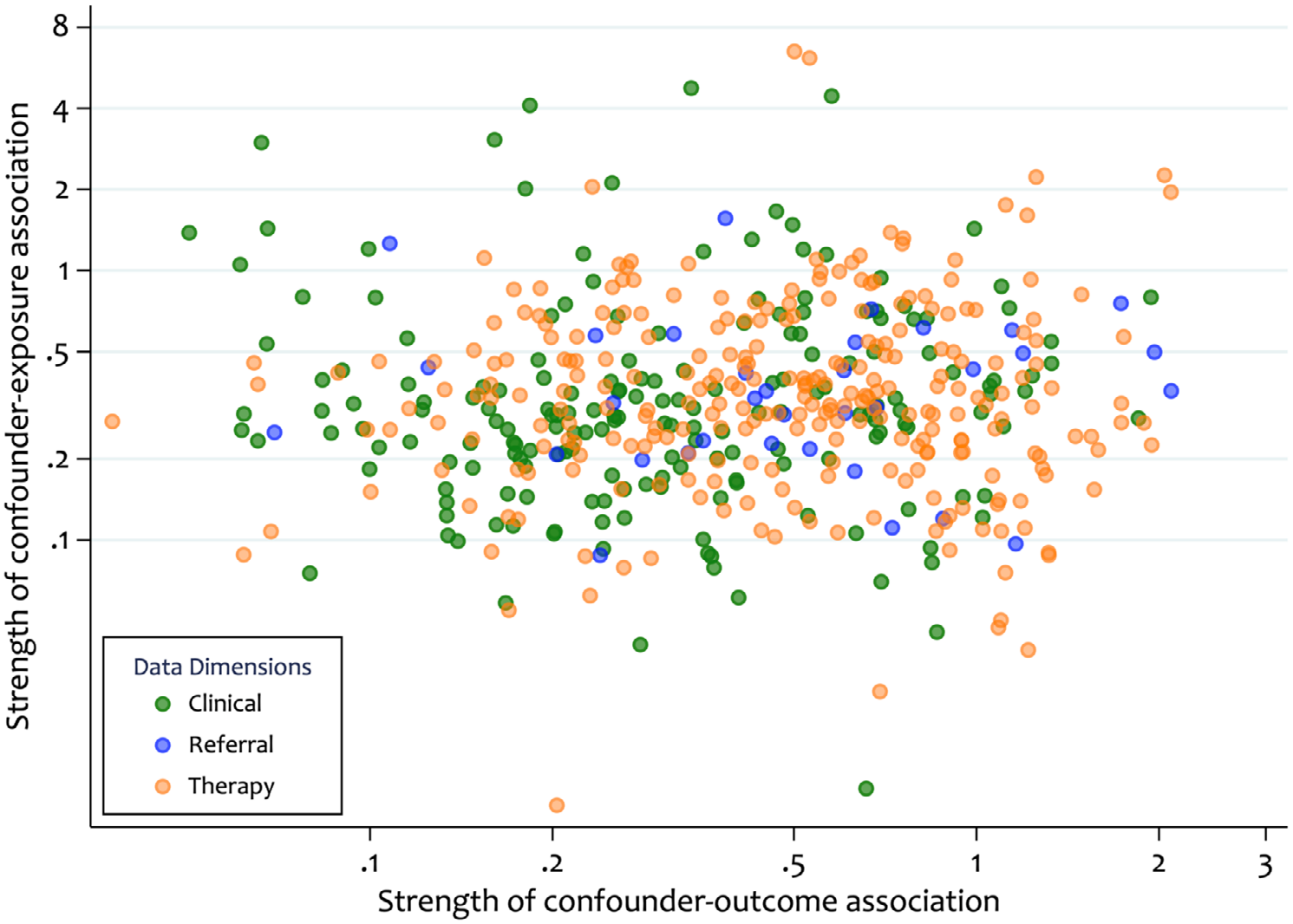
Comparison of the covariate‐exposure and covariate‐outcome associations for the top 500 bias‐based high‐dimensional propensity score pre‐exposure covariates. The values represent the strength of association, defined as the absolute value of the unvariable association minus 1. Larger values indicate a strong association in either direction or a value of zero indicates no association

The data‐driven nature of the HDPS approach does not preclude adjustment for certain variables, such as instrumental variables (IVs) and colliders, which are typically excluded from PS models.^29^, ^30^, ^34^, ^35^ Whilst Step 4 of the HDPS often attempts to down‐weight covariates with these properties (e.g. prioritisation by the Bross formula down‐weights IVs), these variables could inadvertently be included, especially if the total number of covariates available is small relative to the proportion selected. However, the potential reduction in confounding bias from the inclusion of these covariates will often outweigh any increase in bias and variance induced.^30^, ^34^, ^36^ Whilst there are no statistical tests for classifying these types of variables, we can attempt to identify covariates, which behave empirically like IVs. For this purpose, we define a likely IV or near‐IV as a variable, which is strongly associated with exposure but has a weak association with the outcome.^26^ Figure 8 describes the relationship between the covariate‐exposure and covariate‐outcome associations; covariates in the top‐left quadrant represent those behaving empirically as IVs. The following empirical cut offs have been proposed to identify covariates behaving like IVs: $\left|\log({\mathrm{RR}}_{\mathrm{CE}}\right)\left|>1.5\;\text{and}\;\left|\log({\mathrm{RR}}_{\mathrm{CD}}\right)\right|<0.5\;$ and, more restrictively, $\left|\log({\mathrm{RR}}_{\mathrm{CE}}\right)\left|>1.1\;\text{and}\;\left|\log({\mathrm{RR}}_{\mathrm{CD}}\right)\right|<0.5$; where ${\mathrm{RR}}_{\mathrm{CE}}$ and ${\mathrm{RR}}_{\mathrm{CD}}$ are the risk ratios for the covariate‐exposure and covariate‐outcome respectively.^16^

We explore the sensitivity of results to the inclusion of potentially influential covariates in Section 6.2.

## 6 SENSITIVITY ANALYSES

### 6.1 Varying number of covariates selected

A key decision when applying the HDPS surrounds how many covariates to adjust for. Whilst investigators typically choose 200 or 500 variables to augment the pre‐defined covariates, this is largely a result of convention. Simulation studies in moderate to large samples by Rassen et al suggest that adjusting for approximately 300 HDPS variables is likely to be sufficient.^5^

In practice, precisely how many HDPS variables to adjust for is likely to be dependent on the question of interest, rarity of outcome and the richness of data available in the database under investigation. Furthermore, previous studies indicate that in settings with few outcome events results can vary greatly depending on the number of covariates selected.^4^, ^17^

Machine learning approaches have been proposed to determine the number of covariates selected for adjustment, but these have not yet been widely adopted.^15^, ^16^, ^17^, ^28^ Investigators are usually agnostic about how many covariates to select and therefore should assess the sensitivity of results to this decision.

**FIGURE 9:**
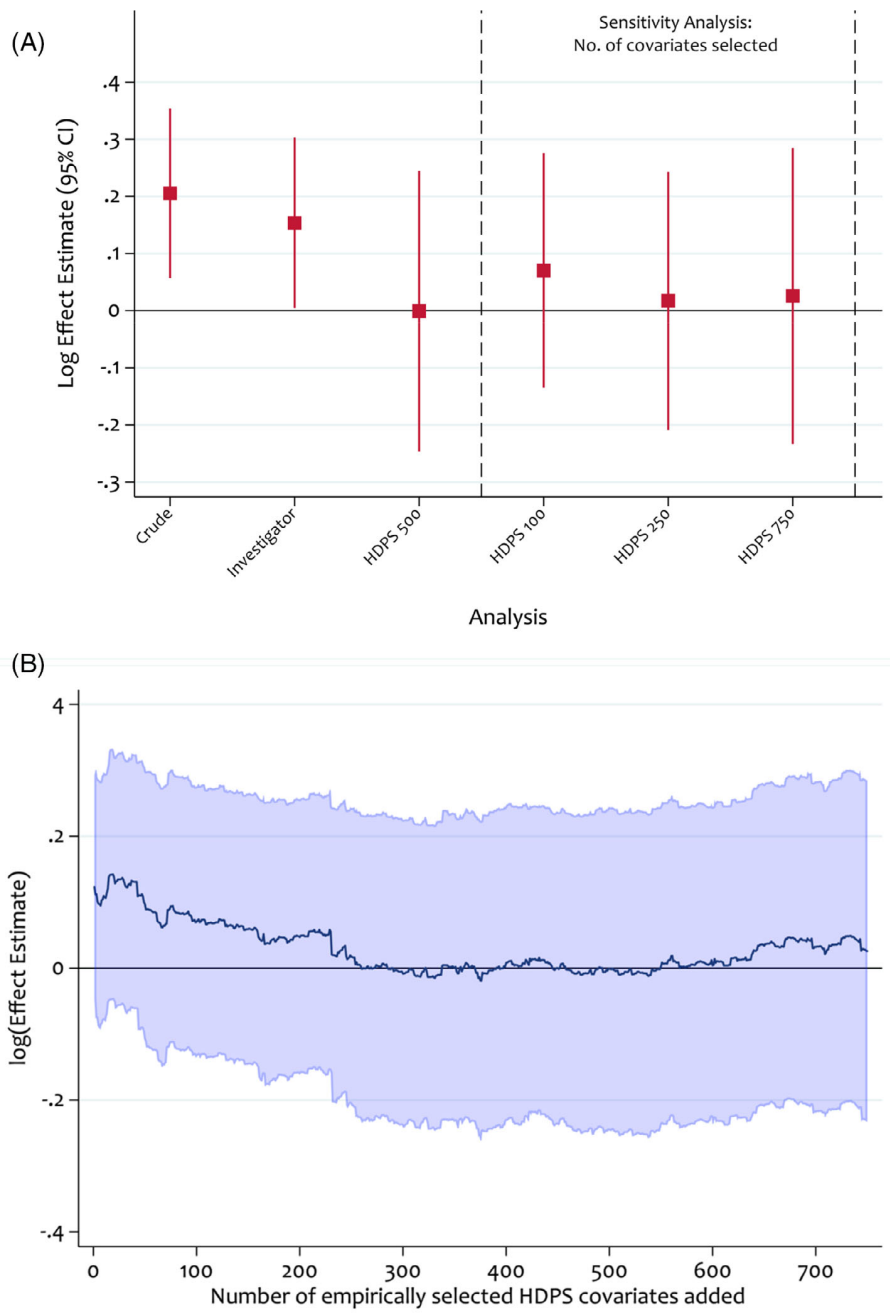
Sensitivity analyses assessing the impact of the number of high‐dimensional propensity score covariates selected on the log effect estimate. Propensity scores were estimated using logistic regression and treatment effects were estimated using an inverse probability of treatment weighted Cox model

Figure 9 presents two options for varying the number of covariates selected. The first specifies a discrete number of scenarios, for example, a study selecting 500 covariates in the primary analysis might investigate the results obtained from selecting 100, 250 and 750 covariates. Figure 9A presents these results next to the primary HDPS analysis, crude model and pre‐defined covariates model. Compared to the crude and investigator analysis, varying the number of HDPS covariates selected resulted in consistent, but not monotonic, shifts in our point estimate towards the expected null association.

Another approach investigates the impact of incrementally adjusting for the empirically selected variables (Figure 9B).^4^ Figure 9B indicates stabilised results with the inclusion of between 250 and 600 covariates. Where results do not stabilise, investigators should try to understand the driving factors and avoid undue focus on a specific HDPS analysis. Instead, it may be more suitable to report a range of effect estimates.

### 6.2 Quantifying impact of potentially influential covariates

In this section we quantify the impact of potentially influential covariates on results obtained in our primary analysis.

The distribution of Bross values (Figure 7) highlights that the top three ranked HDPS covariates are modestly higher than the rest. To understand the extent to which these covariates explain changes in the point estimates after inclusion of HDPS covariates, we conducted a sensitivity analysis adjusting for the predefined covariates plus only the top three ranked covariates (Table 5). We obtained a HR of 1.12 (95% CI: 0.93 to 1.34), indicating some residual confounding remained compared to adjustment for the full set of 500 HDPS covariates (HR 1.00; 95% CI: 0.78–1.28).

**TABLE 5:** Sensitivity analyses exploring the impact of identified potentially influential covariates

Sensitivity type	Sensitivity conditions	Number of covariates removed	Total number of HDPS covariates	Hazard ratio (95% CI)	Confidence limit ratio
Demographics and predefined only	–	–	–	1.17 (1.00–1.35)	1.35
Primary HDPS	–	–	500	1.00 (0.78–1.28)	1.64
Empirical	Pick the top 3 Bross ranked	497	3	1.12 (0.93–1.34)	1.44
	$\left|\log\left({\mathrm{RR}}_{\mathrm{CE}}\right)\right|>1.5$ and $\left|\log\left({\mathrm{RR}}_{\mathrm{CD}}\right)\right|<0.5$	4	496	1.06 (0.87–1.30)	1.49
	$\left|\log\left({\mathrm{RR}}_{\mathrm{CE}}\right)\right|>1.1$ and $\left|\log\left({\mathrm{RR}}_{\mathrm{CD}}\right)\right|<0.5$	9	491	1.06 (0.89–1.26)	1.42
Graphically assess	Figure 8	7	493	1.06 (0.86–1.30)	1.51

In Section 5.3 we identified covariates that behave empirically like IVs. To test the sensitivity of results to their inclusion, we conducted analyses based on Figure 8 (removing seven near‐IVs) and the two cut‐offs previously described. Removing empirically identified IVs altered results in the 2nd decimal point only, indicating no change in the overall interpretation (Table 5). Furthermore, removal of the empirical near‐IV variables resulted in reduced variance around the treatment effect estimate compared to the primary HDPS analysis (Table 5).

## 7 DISCUSSION

The HDPS approach has become a popular and scalable method for augmenting confounder adjustment in a given data source.^10^ However, as with PS analyses more generally, use of diagnostics and reporting of the details of the implementation is suboptimal.^37^, ^38^ Using data from the UK CPRD^14^, ^20^ we highlighted diagnostic tools for assessing HDPS models and proposed considerations for reporting key features.

Drawing on established PS methodology, we described the importance of inspecting the estimated PS distributions before and after inclusion of the HDPS covariates. We recommended assessing covariate balance on important key confounders before and after inclusion of the HDPS covariates to investigate the potential impact of adjusting for many covariates on a set of strong confounders. Additionally, we described diagnostic tools more specific to the HDPS setting, for example, for identifying instrumental‐like variables and informing sensitivity analyses surrounding influential covariates.

We recommend that thorough sensitivity analyses should be conducted and reported when applying the HDPS. A key issue surrounds the number of covariates selected for inclusion in the PS model,^4^, ^17^ especially since the optimal number in a given setting is often unknown. Where inconsistencies are found, efforts should be made using the tools described to understand the drivers of variability.

HDPS covariate prioritisation is often based on univariable associations (e.g., via the Bross formula) and this can potentially lead to the inclusion of covariates which conditionally are not confounders. This has motivated recent developments focussing on the refinement of covariate prioritisation and selection within the HDPS procedure, especially using machine‐learning methods.^15^, ^17^, ^28^, ^39^ Whilst such developments can potentially improve HDPS analyses, no single approach is always optimal and applying the diagnostic tools described here is important to better understand the differences between these approaches.

We hope reporting of these analyses may be improved through more widespread use of the considerations and tools presented here.

## CONFLICT OF INTEREST

Ian J. Douglas has received grants from GlaxoSmithKline, ABPI and NIHR for projects unrelated to the submitted work and owns shares in GlaxoSmithKline. Liam Smeeth has received grants from GlaxoSmithKline for unrelated work. Shirley V. Wang has received salary support from investigator‐initiated grants to Brigham and Women's Hospital from Novartis, Boehringer Ingelheim, and Johnson & Johnson for unrelated work. Sebastian Schneeweiss is a consultant to Aetion, Inc., a software manufacturer in which he owns equity. He is the principal investigator of investigator‐initiated grants to the Brigham and Women's Hospital from Boehringer Ingelheim unrelated to the topic of this study. Joshua J. Gagne has received salary support from grants from Eli Lilly and Company and Novartis Pharmaceuticals Corporation to Brigham and Women's Hospital and was a consultant to Optum, Inc., all for unrelated work. Richard Wyss has received funding from UCB pharma for unrelated work and has consulted for Aetion Inc.

## ETHICS STATEMENT

Scientific approval was obtained to use CPRD data by the Independent Scientific Advisory Committee (ISAC) (Protocol 17_194) and ethical approval from the London School of Hygiene & Tropical Medicine ethics committee.
